# Association between sleep quality and psychological symptoms: A cross-sectional survey of Chinese university students performed during the COVID-19 pandemic

**DOI:** 10.3389/fpsyg.2023.1131176

**Published:** 2023-05-11

**Authors:** Yanyan Hu, Jingzhi Liu, Zhimin Zhao, Cunjian Bi, Hongmin Cao, He Liu, Guangyan Yang

**Affiliations:** ^1^Research Department of Physical Education, Xinjiang Institute of Engineering, Urumqi, China; ^2^School of Physical Education, Chizhou University, Chizhou, China; ^3^Sports Health Promotion Center, Chizhou University, Chizhou, China; ^4^Research Department of Physical Education, Xinjiang University, Urumqi, China

**Keywords:** association, sleep quality, psychological symptoms, university students, COVID-19 pandemic

## Abstract

**Background:**

Since the start of the coronavirus 2019 pandemic, people have faced many challenges, including in relation to sleep quality and psychological health. This study aims to analyze the association between sleep quality and psychological symptoms among university students in China, and to provide reference data to facilitate the development of interventions to improve the physical and mental health of university students.

**Methods:**

A stratified cluster sampling method was used to investigate the sleep quality and psychological symptoms of 6,363 university students in China. The Chi-square test was used to analyze differences in sleep quality among groups. Logistic regression analysis was used to analyze the association between sleep quality and psychological symptoms.

**Results:**

The proportions of Chinese university students with good, medium, and poor sleep quality were 25.73, 10.99, and 63.28%, respectively. The overall rate of psychological symptoms was 16.5%. Logistic regression analysis showed that, in general, university students with poor sleep quality had a higher risk of psychological symptoms than those with good sleep quality (*OR* = 1.53, 95%CI: 1.28, 1.84, *p* < 0.01). Compared with university students with good sleep quality, those with poor sleep quality were more likely to experience emotional symptoms (*OR* = 1.62, 95%CI: 1.36, 1.94), behavioral symptoms (*OR* = 1.55, 95% *CI*: 1.3, 1.84), and difficulties with social adaptation (*OR* = 1.84, 95% *CI*: 1.51, 2.25) (all *p* < 0.01).

**Conclusion:**

There was an association between sleep quality and psychological symptoms among Chinese university students. University students with poor sleep quality have a higher risk of psychological symptoms. Measures should be taken to improve the sleep quality of university students and reduce the incidence of psychological symptoms. This study provides reference data for government and education departments that could inform public health policies.

## 1. Introduction

Sleep plays a very important role in health (Irwin, 2015). Sleep has a major impact on the innate immune system (Irwin and Opp, 2017), inflammation (Irwin, 2019), occurrence and development of major diseases such as cancer (Garland et al., 2018), cardiovascular diseases (Drager et al., 2017), and dementia (Shi et al., 2018), and mental health (Alonzo et al., 2021). However, many people experience sleep disorders; in particular, during the coronavirus 2019 (COVID-19) pandemic, the estimated global prevalence of sleep disorders reached 40.49% (Jahrami et al., 2022). According to the Chinese Sleep Research Report 2022, the sleep time and quality of the population is decreasing every year. Survey data from 2021 showed that 64.75% of Chinese people slept for <8 h a day (Ge et al., 2019). The transition from high school to undergraduate life is a critical period in young people’s lives affected by academic pressure, electronic media use, caffeine intake, and social communication pressure. These factors can lead to sleep problems, which have a significant negative impact on health, study, and employment; inattention, decreased executive function, poor academic performance, obesity, cardiometabolic dysfunction, and even suicidal behaviors may be seen and can continue to impact health in adulthood (Owens and Weiss, 2017; de Zambotti et al., 2018; Dinis and Braganca, 2018; Ge et al., 2019). In summary, sleep quality is of great significance to the health of university students.

Mental health refers to a state in which a person’s cognition, will, emotions, and behavior are in harmony (Shengtao, 2021). University students experience many stressors; various mental health disorders are most likely to onset in this population (Huang et al., 2018). Age, gender, personality, lifestyle, dietary behavior, sleep, physical activity, and screen time are all associated with psychological symptoms in university students (Ge et al., 2019; Bi et al., 2022; Liu et al., 2022; Wang et al., 2022). According to the 2019 Global Burden of Disease, Injury and Risk Factors (GBD) study, the number of people with mental disorders worldwide increased from 80.8 million in 1990 to 125.3 million in 2019, and mental disorders remain among the top 10 disease in terms of the global burden (Collaborators, 2022). The rate of depressive symptoms among Chinese university students after the outbreak of COVID-19 was reported as 35.7% (Zhai et al., 2022). Psychological problems can negatively affect the future development and achievement of university students.

Mood disorders, substance use, circadian rhythm disturbances, and sleep difficulties in university students were significantly associated with depression and anxiety (Samaranayake et al., 2014). Compared with students without insomnia symptoms, those with such symptoms had a higher risk of suicidal ideation, suicide planning, and suicide attempts (de Zambotti et al., 2018). In another study, insomnia was a mediator of paranoia and hallucinations, and a causal factor in the onset of psychotic experiences and other mental health problems (Freeman et al., 2017). In that study, 60% of the improvement in paranoia symptoms was due to adequate sleep quantity and quality, which also led to improvements in depression, anxiety, mental health, nightmares and self-perceived functioning (Freeman et al., 2017). A report on the effects of physical activity, time spent sedentary, and sleep duration on university students’ health-related quality of life showed that students who slept for >9 h per day had significantly superior summary scores for various psychological factors than those who slept for 7–8 h per day, and that increased physical activity and adequate sleep had a positive effect on their health-related quality of life (Ge et al., 2019). To the best of our knowledge, there is a lot of studies on psychological symptoms, but studies on sleep quality and psychological symptoms among Chinese university students are scarce, especially in the context of the COVID-19 pandemic. We have previously only looked at depression, and did not include other mental health problems (Bi et al., 2022).

With the increasing employment pressure experienced by university students, the onset and exacerbation of sleep problems, anxiety, and depression may increase (Chen et al., 2022). During the COVID-19 pandemic, 69.0 and 73.5% of male and female Chinese university students had poor sleep quality, and the rates of depressive symptoms were 43.6 and 47.8%, respectively (Bi et al., 2022). Another meta-analysis showed that one in four Chinese college students had symptoms of anxiety during the COVID-19 pandemic, with a higher prevalence of anxiety in the later stages of the pandemic compared with the earlier stages (29.1% vs. 17.2%) (Wang et al., 2022). Because sleep and psychiatric disorders among university students in China are becoming increasingly problematic. However, through literature search, we found that although there are many studies on the psychological symptoms and sleep problems of Chinese university students, there are few articles that study the relationship between the two. So it is necessary to study the association between sleep quality and psychological symptoms in this population. Therefore, this study investigated the association of sleep quality with psychological symptoms among 6,363 university students in China; the goal was to find ways to improve the quality of sleep and promote mental health in university students and provide reference data that could aid decision-making by educational and public health departments.

## 2. Materials and methods

### 2.1. Participants

**Figure 1:**
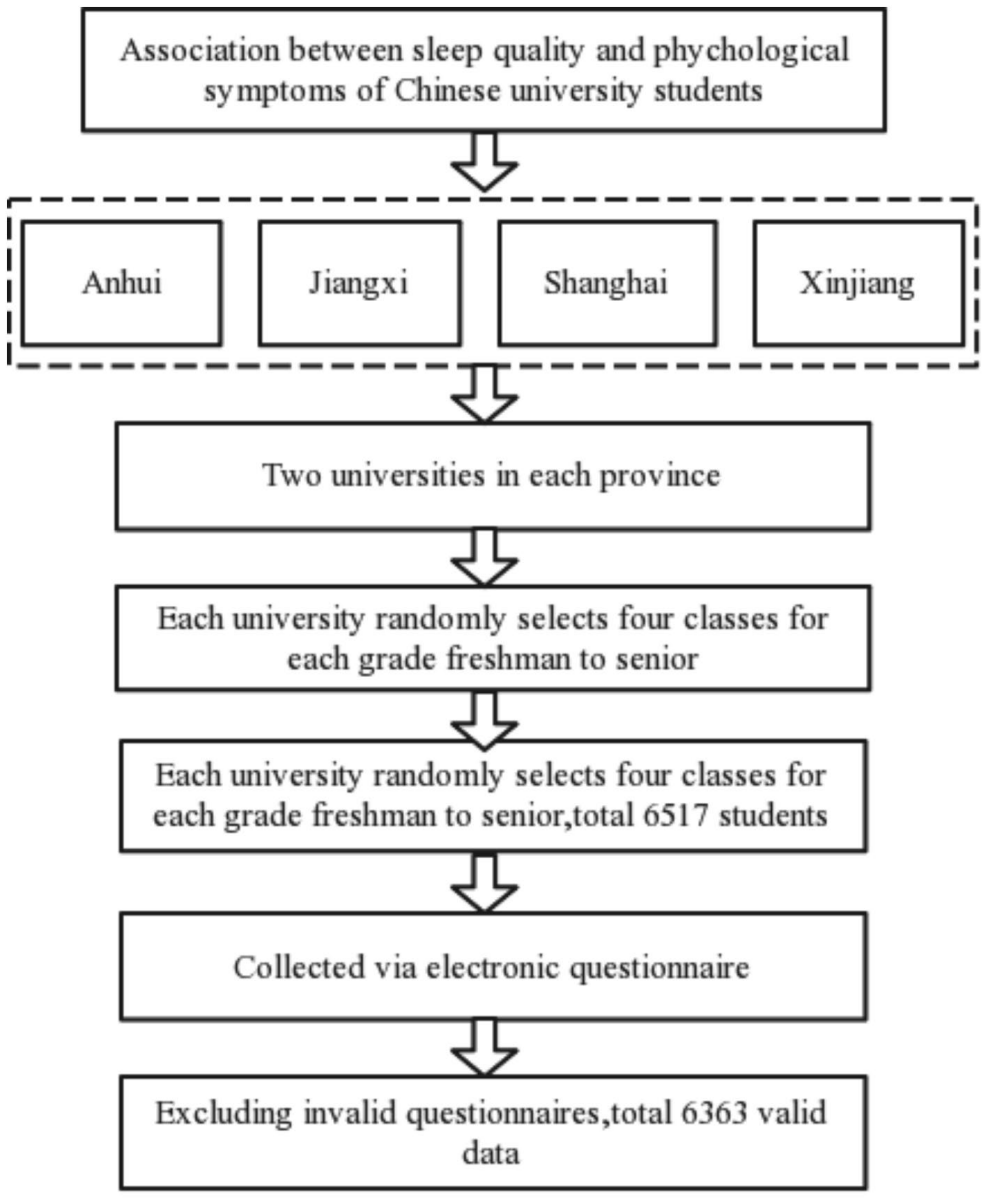
Sampling process of the association between sleep quality and psychological symptoms among Chinese university students.

Participants were obtained through stratified cluster sampling in three stages: First of all, according to the geographic distribution of Chinese provinces, this study selects Anhui, Jiangxi, Shanghai and Xinjiang as the research area. Secondly, two universities in each province were selected as the survey universities in this study. Third, from freshman to senior year in each university, four teaching classes were randomly selected from each class as the sampling unit. A total of 6,517 college students from 128 teaching classes in 8 universities were selected in this study. After excluding invalid questionnaires, a total of 6,363 valid data were collected (2,745, 43.1% of male students), and the effective data recovery rate was 97.64%. The survey period was September–December 2021 (Figure 1).

### 2.2. Data collection

In this study, data were mainly collected *via* an electronic questionnaire. The investigators were faculty members and graduate students. To ensure data validity, the investigators attended a training course before collecting survey data. Moreover, the purpose, requirements and precautions of the study were explained to the subjects in a standardized manner. Then, the investigators distributed QR codes to the subjects and asked them to scan the code and fill in the electronic questionnaire. Students use their smartphone scan the code and fill in the electronic questionnaire. Investigators were present throughout the questionnaire completion and submission process. The questionnaire included basic questions about the subjects, the Pittsburgh Sleep Quality Index (PSQI), and a Psychological Health of Youths (BIOPHY), among other components. The basic questions including age, gender, grade, college, parents’ education.

Written informed consent was obtained from the students prior to the survey. This study was carried out with approval from the Human Ethics Committee of Chizhou University (202104089).

### 2.3. Sleep quality survey

The sleep quality survey used in this study was the PSQI scale. The PSQI is the most commonly used subjective measure of sleep quality, and has been proven to have good reliability and validity (Tsai et al., 2005; Guo et al., 2016; Mollayeva et al., 2016). The average of PSQI scores was 5.38 ± 2.34, the total Cronbach’s α coefficient of all the scale items was 0.734,and the half-split reliability coefficient was 0.655 (Zheng et al., 2016). PWe used the PSQI to investigate sleep quality and disorders in the past month. The PSQI has 18 items distributed among seven factors: subjective sleep quality, sleep latency, duration of sleep at night, sleep efficiency, sleep disorders, use of hypnotic drugs, and daytime dysfunction. The maximum score on the PSQI score is 21 points, and higher scores indicate poorer sleep quality. Total scores ≦5 indicate good sleep quality, scores of 6–7 denote moderate quality, and scores >7 reflect poor sleep quality.

### 2.4. Psychological symptoms

For the investigation of psychological symptoms, we used the Brief Instrument on Psychological Health of Youths (BIOPHY) developed by Professor Tao Fangio’s team. The Brief Instrument on Psychological Health of Youths (BIOPHY) is a simplified version of the Multidimensional Sub-health Questionnaire of Adolescents (MSQA). The Cronbach’s α coefficient of MSQA total questionnaire score was 0.961, and the split half reliability coefficient was 0.938, and the statistical results for BIOPHY were as follows: Kaiser–Meyer–Olkin statistic, 0.953; Cronbach’s alpha, 0.928; and split-half coefficient, 0.909. On the basis of these data, BIOPHY has been confirmed that it meets the psychological evaluation standard and has good predictive validity and can be popularized and used in the research of adolescent mental health in the future (Tao et al., 2020). BIOPHY has 15 items distributed among three dimensions (emotional symptoms, behavioral symptoms, and difficulties with social adaptation). Items 1–7 pertain to emotional symptoms (e.g., “I am not interested in things,” “Often feel nervous,” “Often blame yourself,” “Often feel restless and uneasy,” “Often hesitate to do things,” “Often feel miserable,” “Unnecessary thoughts are always in the mind”). Items 8, 10, 11, and 13 are concerned with behavioral symptoms (e.g., “I always feel that others are against me,” etc.). Items 9, 12, 14, and 15 pertain to difficulties with social adaptation (e.g., “I feel that most people cannot be trusted,” etc.). Each item is reverse-scored and the response options are as follows: (1) Has been true for more than 3 months; (2) Has been true for than 2 months; (3) Has been true for more than 1 month; (4) Has been true for more than 2 weeks; (5) Has been true for more than 1 week; and (6) Not true or true for less than 1 week. Higher scores indicate a shorter duration of psychological symptoms. One point is awarded for response options 1–3, and 0 points for options 4–6. A total score in the 90th quantile (score of ≥7; maximum possible score = 15) was considered to indicate the presence of psychological symptoms in adolescents. Regarding the individual dimensions, a score of ≥4 indicates the presence of emotional symptoms, while a score of ≥1 indicates the presence of behavioral symptoms, and a score of ≥2 difficulties with social adaptation.

### 2.5. Covariates

Covariates included sex, father’s education level, mother’s education level, screen time, body mass index (BMI), and moderate-to-vigorous physical activity (MVPA). Father’s and mother’s education level was classified as primary school or below, secondary or high school, or junior university or above. Screen time was classified as ≧2 h or <2 h. BMI was calculated as weight (kg)/height (m)^2^ and divided into four categories: ≦18.4, underweight; 18.5–23.9, normal weight; 24.0–27.9, overweight, and ≧28, obese. The height and weight were measured according to the China National Student Physical Fitness Standards 2014 edition, to within 0.1 cm and 0. 1 kg, respectively. The China National Student Physical Fitness Standards 2014 edition had been promulgated and modified by the Ministry of Education of China. It has been proved to be more scientific and proved to be more scientific, with clear dimensions and comprehensive observation (CNSSCH Association, 2016). The MVPA was assessed on the basis of the physical activity section of the China National Student Physical Fitness Survey. The average frequency and duration of participation in MVPA per day over the past 7 days were calculated to derive daily MVPA values. Examples of MVPA include sprinting, ball games, skiing, cycling, etc. MVPA was classified by percentile (<30%, bottom percentile; 30–70%, middle percentile; >70%, top percentile).

### 2.6. Statistical analysis

Categorical sleep quality variables are presented as numbers and percentages, and were analyzed using the Chi-square test. Psychological symptoms were analyzed according to sleep quality using the Chi-square test and logistic regression. Three models (crude mode and models 1 and 2) were constructed to analyze the associations of sleep quality with emotional symptoms, behavioral symptoms, difficulties with social adaptation, and psychological symptoms. Model 1 controlled for age, father’s education level, and mother’s education level. Model 2 additionally controlled for screen time, BMI, and MVPA. Data were analyzed using SPSS software (ver. 25.0; IBM Corp., Armonk, NY, United States). An α value of 0.05 was considered significant.

## 3. Results

The total number of subjects in this study was 6,363, 2,745 (43.1%) of whom were male. The mean age of the subjects was (21.12 ± 1.17) years. The proportions of students with good, moderate, and poor sleep quality were 25.73, 10.99, and 63.28%, respectively.

Sleep quality differed according to sex, father’s education, mother’s education, screen time, and amount of MVPA (*χ*^2^ = 69.046, 46.294, 57.733, 689.068, and 29.665, respectively; all *p* < 0.001). The likelihood of poor sleep quality was higher for females, as well as for students whose father or mother had a high school-level education, and students with a screen time ≥2 h/d and low amount of MVPA (Table 1).

**Table 1:** Sleep quality (PSQI) scores of university students in China.

Characteristics	PSQI			Total (*N* = 6,363)	*χ*^2^ value	*p*-value
	Good (*n* = 1,637)	Moderate (*n* = 699)	Low (*n* = 4,027)			
**Sex**						
Male	848 (51.8)	296 (42.3)	1,601 (39.8)	2,745 (43.1)	69.046	<0.001
Female	789 (48.2)	403 (57.7)	2,426 (60.2)	3,618 (56.9)		
**Father’s education**						
Primary school or below	389 (23.8)	178 (25.5)	1,243 (30.9)	1,810 (28.4)	46.294	<0.001
Secondary or high school	1,081 (66.0)	445 (63.7)	2,500 (62.1)	4,026 (63.3)		
Junior university or above	167 (10.2)	76 (10.9)	284 (7.1)	527 (8.3)		
**Mother’s education**						
Primary school or below	669 (40.9)	327 (46.8)	1,928 (47.9)	2,924 (46)	57.733	<0.001
Secondary or high school	848 (51.8)	326 (46.6)	1,960 (48.7)	3,134 (49.3)		
Junior university or above	120 (7.3)	46 (6.6)	139 (3.5)	305 (4.8)		
**Screen time**						
≥2 h/d	814 (49.7)	562 (80.4)	3,343 (83)	4,719 (74.2)	689.068	<0.001
<2 h/d	823 (50.3)	137 (19.6)	684 (17)	1,644 (25.8)		
**BMI (kg/m** ^**2**^ **)**						
Underweight	271 (16.6)	103 (14.7)	672 (16.7)	1,046 (16.4)	10.001	0.125
Normal	876 (53.5)	373 (53.4)	2,121 (52.7)	3,370 (53.0)		
Overweight	202 (12.3)	112 (16.0)	499 (12.4)	813 (12.8)		
Obese	288 (17.6)	111 (15.9)	735 (18.3)	1,134 (17.8)		
**MVPA amount per day**						
Low^*^	1,198 (73.2)	509 (72.8)	3,169 (78.7)	4,876 (76.6)	29.665	<0.001
Medium^**^	343 (21.0)	160 (22.9)	687 (17.1)	1,190 (18.7)		
High^***^	96 (5.9)	30 (4.3)	171 (4.2)	297 (4.7)		

Continuous and categorical data are presented as mean (standard deviation) and number (percentage), respectively. *<30th percentile; **30th–70th percentile; ^***^>70th percentile. h/d, hour/day; BMI, body mass index; MVPA, moderate-to-vigorous physical activity.

As shown in Table 2, the overall rate of psychological symptoms was 16.5% (*n* = 1,049/6,363); the rate was 14.8% (*n* = 407/2,745) in males and 17.7% (*n* = 642/3,618) in females. Compared with university students with moderate or good sleep quality, those with poor sleep quality had higher rates of psychological, emotional and behavioral symptoms, and a higher likelihood of difficulties with social adaptation (19.8, 21.5, 21.7, and 18.3%; *χ*^2^ = 87.445, 106.543, 98.958, and 125.243, respectively; all *p* < 0.001) (Table 2).

**Table 2:** Sleep quality (PSQI) scores according to psychological symptoms among university students in China (*N* = 6,363).

Psychological symptoms	PSQI	*N*	%	*χ*^2^ value	*p*-value
**Males**					
Emotional symptoms	Good	73	8.6	81.817	<0.001
	Moderate	24	8.1		
	Poor	341	21.3		
Behavioral symptoms	Good	79	9.3	72.859	<0.001
	Moderate	29	9.8		
	Poor	348	21.7		
Difficulties with social adaptation	Good	63	7.4	84.554	<0.001
	Moderate	19	6.4		
	Poor	315	19.7		
Psychological symptoms	Good	67	7.9	73.364	<0.001
	Moderate	24	8.1		
	Poor	316	19.7		
**Females**					
Emotional symptoms	Good	125	15.8	34.005	<0.001
	Moderate	43	10.7		
	Poor	525	21.6		
Behavioral symptoms	Good	129	16.3	35.815	<0.001
	Moderate	40	9.9		
	Poor	525	21.6		
Difficulties with social adaptation	Good	77	9.8	47.016	<0.001
	Moderate	29	7.2		
	Poor	420	17.3		
Psychological symptoms	Good	121	15.3	25.886	<0.001
	Moderate	41	10.2		
	Poor	480	19.8		
**Entire cohort**					
Emotional symptoms	Good	198	12.1	106.543	<0.001
	Moderate	67	9.6		
	Poor	866	21.5		
Behavioral symptoms	Good	208	12.7	98.958	<0.001
	Moderate	69	9.9		
	Poor	873	21.7		
Difficulties with social adaptation	Good	140	8.6	125.243	<0.001
	Moderate	48	6.9		
	Poor	735	18.3		
Psychological symptoms	Good	188	11.5	87.445	<0.001
	Moderate	65	9.3		
	Poor	796	19.8		

PSQI, Pittsburgh Sleep Quality Index.

After adjusting for age, father’s education level, mother’s education level, screen time, BMI, and MVPA (model 2), multivariate logistic regression analysis showed that university students with poorer sleep quality had a higher risk of developing psychological symptoms compared with those with good sleep quality (*OR* = 1.53, 95%*CI*: 1.28, 1.84, *p* < 0.01). Compared with university students with good sleep quality, those with poor sleep quality had a higher risk of developing emotional symptoms (*OR* = 1.62, 95%*CI*: 1.36, 1.94) and behavioral symptoms (*OR* = 1.55, 95%*CI*: 1.3, 1.84), and were also more likely to experience difficulties with social adaptation (*OR* = 1.84, 95%*CI*: 1.51, 2.25) (all *p* < 0.01). The effect of poor sleep quality on psychological symptoms was more significant for male university students (*OR* = 2.20, 95%*CI*: 1.64, 2.95) (*p* < 0.01), while it did not have a significant effect on female students (*OR* = 1.13, 95%*CI*: 0.89, 1.43) (*P* > 0.05) (Table 3).

**Table 3:** Multiple logistic regression analysis of sleep quality and psychological symptoms among Chinese university students (*N* = 6,363).

Psychological symptoms	PSQI	Odds ratio (95% confidence interval)		
		Crude model	Model 1	Model 2
**Males**				
Emotional symptoms	Good	1.00	1.00	1.00
	Moderate	0.94 (0.58, 1.52)	0.99 (0.61, 1.61)	0.85 (0.52, 1.39)
	Poor	2.87 (2.20, 3.76)^b^	2.71 (2.07, 3.56)^b^	2.25 (1.7, 2.98)^b^
Behavioral symptoms	Good	1.00	1.00	1.00
	Moderate	1.06 (0.68, 1.65)	1.12 (0.72, 1.76)	0.97 (0.61, 1.52)
	Poor	2.70 (2.08, 3.51)^b^	2.58 (1.99, 3.36)^b^	2.14 (1.63, 2.81)^b^
Difficulties with social adaptation	Good	1.00	1.00	1.00
	Moderate	0.86 (0.50, 1.45)	0.92 (0.54, 1.56)	0.75 (0.43, 1.28)
	Poor	3.05 (2.30, 4.06)^b^	2.90 (2.18, 3.87)^b^	2.25 (1.67, 3.03)^b^
Psychological symptoms	Good	1.00	1.00	1.00
	Moderate	1.03 (0.63, 1.67)	1.10 (0.67, 1.79)	0.91 (0.56, 1.51)
	Poor	2.87 (2.17, 3.79)^b^	2.71 (2.05, 3.59)^b^	2.20 (1.64, 2.95)^b^
**Females**				
Emotional symptoms	Good	1.00	1.00	1.00
	Moderate	0.63 (0.44, 0.92)^a^	0.57 (0.39, 0.83)^b^	0.51 (0.35, 0.76)^b^
	Poor	1.47 (1.18, 1.82)^b^	1.36 (1.09, 1.69)^a^	1.21 (0.96, 1.53)
Behavioral symptoms	Good	1.00	1.00	1.00
	Moderate	0.56 (0.39, 0.82)^b^	0.52 (0.35, 0.76)	0.45 (0.31, 0.67)^b^
	Poor	1.41 (1.14, 1.75)^b^	1.32 (1.06, 1.63)	1.16 (0.92, 1.45)
Difficulties with social adaptation	Good	1.00	1.00	1.00
	Moderate	0.72 (0.46, 1.12)	0.67 (0.43, 1.05)	0.57 (0.36, 0.90)^a^
	Poor	1.94 (1.50, 2.51)^b^	1.84 (1.42, 2.38)^b^	1.55 (1.18, 2.03)^b^
Psychological symptoms	Good	1.00	1.00	1.00
	Moderate	0.63 (0.43, 0.91)^a^	0.57 (0.39, 0.83)^b^	0.51 (0.34, 0.76)^a^
	Poor	1.36 (1.10, 1.69)^b^	1.27 (1.01, 1.58)^a^	1.13 (0.89, 1.43)
**Total**				
Emotional symptoms	Good	1.00	1.00	1.00
	Moderate	0.77 (0.58, 1.03)	0.76 (0.57, 1.02)	0.66 (0.49, 0.89)^a^
	Poor	1.99 (1.69, 2.35)^b^	1.89 (1.6, 2.24)^b^	1.62 (1.36, 1.94)^b^
Behavioral symptoms	Good	1.00	1.00	1.00
	Moderate	0.75 (0.56, 1.00)	0.74 (0.56, 0.99)^a^	0.64 (0.48, 0.86)^b^
	Poor	1.90(1.62, 2.24)^b^	1.81 (1.53, 2.14)^b^	1.55 (1.30, 1.84)^b^
Difficulties with social adaptation	Good	1.00	1.00	1.00
	Moderate	0.79 (0.56, 1.11)	0.78 (0.56, 1.10)	0.64 (0.45, 0.91)^a^
	Poor	2.39 (1.97, 2.89)^b^	2.28 (1.88, 2.76)^b^	1.84 (1.51, 2.25)^b^
Psychological symptoms	Good	1.00	1.00	1.00
	Moderate	0.79 (0.59, 1.06)	0.78 (0.58, 1.05)	0.67 (0.50, 0.91)^a^
	Poor	1.90 (1.6, 2.25)^b^	1.81 (1.52, 2.15)^b^	1.53 (1.28, 1.84)^b^

Model 1 controlled for age, father’s education level, and mother’s education level; model 2 also controlled for screen time, body mass index, and moderate-to-vigorous physical activity. ^a^*P* < 0.05, ^b^*P* < 0.01. PSQI, Pittsburgh Sleep Quality Index.

## 4. Discussion

In total, 63.28% of the Chinese university students in this experienced relatively poor sleep quality during the COVID-19 pandemic, consistent with the findings of the China Sleep Research Report 2022. Less educated parents may have less time to raise their children. Moreover, longer screen times are associated with less leisure and exercise activities, and poorer sleep quality (Jeon and Kim, 2022). Screen time is increasing in university students, especially before going to sleep; this can delay sleep and affect melatonin secretion, thus leading to a gradual decline in sleep (Royant-Parola et al., 2018). Numerous studies have shown that more MVPA is associated with better sleep quality in university students (Memon et al., 2021). Taken together, increasing the companionship time by parents, reasonably controlling screen time <2 h, and encouraging to increase the time spent participating in MVPN every day can effectively improve the sleep quality of college students.

The rate of psychological symptoms among our Chinese university students during the COVID-19 pandemic was 16.5%, which is lower than in a previous study (35.7%) (Zhai et al., 2022). This may be due to differences in the criteria used to evaluate psychological symptoms. Huang et al. (2022) found that the prevalence of psychological symptoms among adolescents was lower after the COVID-19 pandemic, which may be attributable to a reduction in bullying due to reduced school hours, increased time spent with parents because of isolation rules, less academic stress, etc. (Huang et al., 2022). Similarly, a US study reported that COVID-19-related isolation policies protected adolescents’ mental health (Penner et al., 2021). Nevertheless, the prevalence of psychological symptoms in our cohort was still higher compared with the rate reported pre-pandemic among university students (14.2%) (Hou et al., 2018). Therefore, psychological symptoms should be the focus of future research. The rate of psychological symptoms was lower in the male (14.8%) than female (17.7%) students in this study. Even before the COVID-19 pandemic, many studies demonstrated a higher prevalence of psychological symptoms in women than men (Yang et al., 2013). Physiologically speaking, women are more likely to fluctuate in hormone levels than men, so they are more emotional and emotional than men. In the face of emergencies, stress reactions and emotions such as nervousness, anxiety, and worry are more likely to occur, resulting in a higher prevalence of anxiety and depression than men. Psychologically, women are more sensitive and receptive to cues than men; As a result, empathy is more likely to develop and more susceptible to emotions and events in those around them, resulting in a higher prevalence of anxiety and depression than men (Zhang et al., 2018; Conteh et al., 2022).

After adjusting for relevant covariates, the university students with poorer sleep quality in our study had a higher risk of psychological symptoms, consistent with the findings of most previous studies. COVID-19 lockdowns have altered daily activities, reduced exercise, and increased stress and anxiety have led to reduced physical health and sleep quality (Dergaa et al., 2022). But there are also studies that have found that overall health and sleep quality during the pandemic were better than normal due to attention to diet, exercise, or physical activity (Dergaa et al., 2022). The reason for this may be that the psychological characteristics during the quarantine period largely depend on individual behavior and lifestyle. Sleep quality and depressive symptoms are in a bidirectional relationship; more severe depressive symptoms lead to an increased risk of poor sleep quality and vice versa (Dinis and Braganca, 2018). Freeman et al. showed that insomnia is an important factor in psychiatric disorders and psychological symptoms, and that digital cognitive behavioral therapy improved the sleep quality of patients, thus reducing psychological problems such as hallucinations and paranoia (Freeman et al., 2017). Strengthening health education improved the sleep quality of university students and thus helped prevent depression (Freeman et al., 2017). However, university students often lack knowledge about sleep, ignore its importance to physical and mental health, and are reticent to seek help for sleep disorders, which can lead to a decline in sleep quality and various psychological problems. Therefore, promptly guiding university students with poor sleep quality toward psychological help, and improving knowledge of sleep and the application thereof, could improve university students’ sleep quality and reduce the likelihood of psychological problems.

Our study had some limitations. First, it was not possible to analyze causal associations between psychological symptoms and sleep quality because we used a cross-sectional design. Second, the subjects’ reports of psychological symptoms could have been affected by recall bias. Third, we did not controlling over the diary behavior of the study population. However, our study also had certain strengths, including a large sample drawn from a wide geographical area (which enhanced representativeness). In addition, our results building on previous studies of the relationship between depression and sleep to explore the relationship between psychological symptoms and sleep and the data could serve as a reference to aid educational and public health decision-making.

## 5. Conclusion

The Chinese university students in this study had poorer sleep quality during the COVID-19 pandemic compared with the pre-pandemic period, which was associated with an increased rate of psychological symptoms. We recommend <2 h of screen time per day, increase the time of MVPN, timely guide college students with poor sleep quality to receive psychological help, and improve college students’ awareness and application of sleep. This could improve university students’ sleep quality and reduce the likelihood of psychological problems. Our data could serve as a reference to aid educational and public health decision-making as it pertains to the problem of psychological symptoms among university students.

## Data availability statement

The raw data supporting the conclusions of this article will be made available by the authors, without undue reservation.

## Ethics statement

The studies involving human participants were reviewed and approved by this study was carried out with approval from the Human Ethics Committee of Chizhou University (202104089). The patients/participants provided their written informed consent to participate in this study.

## Author contributions

YH and CB: conceptualization. HL: data curation and formal analysis. CB: funding acquisition. HC: investigation, methodology, project administration, and software. ZZ: resources and supervision. JL: validation and visualization. YH, CB, and GY: writing—original draft and writing—review and editing. All authors have read and agreed to the published version of the manuscript.

## Funding

Grant for Scientific Research Project of Anhui Provincial Education Department (2022AH051824).

## Conflict of interest

The authors declare that the research was conducted in the absence of any commercial or financial relationships that could be construed as a potential conflict of interest.

## Publisher’s note

All claims expressed in this article are solely those of the authors and do not necessarily represent those of their affiliated organizations, or those of the publisher, the editors and the reviewers. Any product that may be evaluated in this article, or claim that may be made by its manufacturer, is not guaranteed or endorsed by the publisher.
